# Two decades of bacteraemia in Norway: an ecological study of incidence and shifts in microbial epidemiology, 2005–2024

**DOI:** 10.1038/s41598-025-28472-x

**Published:** 2025-12-29

**Authors:** Anders Skyrud Danielsen, Amalie Johansen, Miriam Sare, Cherry Lim, Jørgen Vildershøj Bjørnholt, Anne-Sofie Furberg, Gunnar Skov Simonsen

**Affiliations:** 1 Department of Infection Control and Preparedness, Norwegian Institute of Public Health, Oslo, Norway; 2 Nordland Hospital Vesterålen, Nordland Hospital Trust, Stokmarknes, Norway; 3 Nuffield Department of Medicine, University of Oxford, Oxford, UK; 4 Department of Microbiology, Oslo University Hospital, Oslo, Norway; 5 Institute of Clinical Medicine, University of Oslo, Oslo, Norway; 6 Department of Microbiology and Infection Control, University Hospital of North Norway, Tromsø, Norway; 7 Faculty of Health Sciences and Social Care, Molde University College, Molde, Norway; 8 Research Group for Host-Microbe Interaction, Faculty of Health Sciences, UiT The Arctic University of Norway, Tromsø, Norway

**Keywords:** Bacteraemia, Bloodstream infections, Epidemiology, Surveillance, Microbial distribution, Norway, Diseases, Medical research, Microbiology

## Abstract

This study aimed to describe temporal changes in the incidence and microbial composition of bacteraemia in Norway from 2005 to 2024. Using national surveillance data from all microbiology laboratories, combined with contextual indicators such as population size, hospital activity, immunosuppression, and cancer incidence, we conducted an ecological analysis of annual aggregated data. The number of isolates from blood cultures increased from 10,964 in 2005 to 22,679 in 2024, or from 238.0 to 408.6 per 100,000 people, while the estimated blood culture positivity rate remained stable at approximately 6%. *Escherichia coli*, *Staphylococcus aureus*, and *Klebsiella* spp. were the most frequent pathogens in 2024, while the proportion of *Streptococcus pneumoniae* declined by nearly 40%. The apparent rise in the Gram-negative to Gram-positive ratio was largely explained by this decline in pneumococci. Regression models showed a steady increase in incidence of bacteraemia over time, with minimal impact of age adjustment. Increased diagnostic activity is probably driven by a larger share of inpatients meeting criteria for blood culture, consistent with a frailer and more complex case-mix. The relative stability of estimated positivity rate and the proportion of coagulase-negative staphylococci support that the rise reflects a real increase in the underlying burden of bloodstream infections.

**Supplementary Information:**

The online version contains supplementary material available at 10.1038/s41598-025-28472-x.

## Introduction

The detection of microorganisms in blood cultures indicates a breach in the host’s normal barriers to infection and often signals the presence of a systemic infection. However, a positive blood culture may also reflect contamination or represent a transient bacteraemia effectively contained by the host immune system^1,2^. Typical contaminants include coagulase-negative staphylococci and other skin microbiota. In the Nordic countries, an increase in bacteraemia has been reported^3–5^. Not only is the overall incidence of bacteraemia increasing, but the increase seems to mainly be in Gram-negative bacilli, thereby shifting the underlying microbial epidemiology. The rise is unlikely to reflect a lowering of thresholds for blood culture sampling, since positivity rates have been stable, as shown by Dessau et al.^4^.

Crude counts and rates of blood cultures, with or without contamination, may be misleading if it’s not contextualised by changes in population structure and healthcare activity, such as hospital bed-days, inpatient turnover, and diagnostic testing volume. Several factors may influence temporal trends in bacteraemia. First, demographic changes such as population ageing increase the proportion of individuals at higher risk of invasive infection. This enlarges the population at risk beyond what would be expected from population growth alone and contributes to more blood cultures being taken, though without necessarily raising the positivity rate^4,6^. Second, developments in healthcare—including greater use of immunosuppressive therapies, invasive medical procedures, and cancer treatments—may increase both the number of blood cultures performed and the proportion that are positive^7,8^. Third, changes in microbiological diagnostics—such as modifications in blood culture practices, sampling volumes, or laboratory technology—may increase the number of cultures sampled and, in some cases, also the likelihood of detecting bacteraemia^9^. Understanding these trends is important for public health planning and antimicrobial stewardship, as it provides information about underlying morbidity in the population, supports rational therapeutic strategies, helps identify emerging microbial threats, guide the allocation of resources, and adapt international recommendations to the local context.

In Norway, the aggregate statistics on all positive blood cultures are collected through the Norwegian Surveillance System for Antimicrobial Resistance (NORM), a national programme established in 1999 to monitor antimicrobial resistance in humans^10^. This study aimed to describe temporal changes in the incidence and microbial composition of bacteraemia in Norway between 2005 and 2024 by leveraging the national surveillance data, considering contextual factors such as population age structure, underlying morbidities, and hospital activity.

## Materials and methods

### Data

This ecological study was based on annually aggregated data from national surveillance and health registers in Norway for the years 2005 to 2024. The time period was chosen because by 2005 blood culture statistics had been recorded in their current format, and 2024 represented the most recent year with available data.

National standards for drawing blood cultures to detect bacteraemia in systemic infections have been in place for several decades^11^, and the technical procedures are also uniform across the country with only minor variations. Data on isolates from positive blood cultures were obtained from NORM. NORM collects annual reports from all clinical microbiology laboratories in Norway, using a standardised protocol that includes both the total number of blood culture isolates and the distribution of categories of species. Furthermore, data were deduplicated at the local laboratory level using the national unique personal identifier, including only the first isolate of a given species category per patient within a 30-day window. Subsequent isolates of the same species category during this period were excluded, whereas a new isolate after 30 days is considered a new case and thus included. The dataset includes all microbial findings, including those commonly considered contaminants. The data did not include patient-level information such as age or sex.

In order to calculate the positivity rate (calculation detailed below), we included an indicator for the number of blood cultures taken by collecting data on the aerobic bottles drawn, which served as a proxy for the total number of blood culture sets, as each set in Norway routinely includes one aerobic bottle. Data for this indicator were gathered from seven hospitals from Northern Norway Regional Health Authority (University Hospital of North Norway (UNN) Tromsø), Central Norway Regional Health Authority (St. Olavs Hospital, Levanger Hospital, Molde Hospital, Ålesund Hospital), and South-Eastern Norway Regional Health Authority (Oslo University Hospital Rikshospitalet and Oslo University Hospital Ullevål). These hospitals were selected based on data availability but together represent cross-sections of typical Norwegian hospitals. Corresponding catchment area population data for Central Norway and Northern Norway were obtained from Statistics Norway to support normalisation and estimation.

Statistics on contextual indicators were retrieved from the publicly available databases of Statistics Norway, the Cancer Registry of Norway, and the Norwegian Prescribed Drug Registry^12–14^. In addition, we obtained aggregate statistics from the Norwegian Patient Registry through an application to *Helsedataservice* customised specifically for this study^15^.

### Outcome

All blood culture isolates were classified into one of 21 mutually exclusive species categories:

- *Staphylococcus aureus*,
- Coagulase-negative staphylococci,
- *Streptococcus pneumoniae*,
- *Streptococcus pyogenes*,
- *Streptococcus agalactiae*,
- *Streptococcus dysgalactiae,*
- Viridans and non-haemolytic streptococci,
- *Enterococcus faecalis*,
- *Enterococcus faecium*,
- Other Gram-positive aerobic and facultative anaerobic bacteria,
- *Escherichia coli*,
- *Klebsiella* spp.,
- *Enterobacter* spp.,
- *Proteus* spp.,
- Other *Enterobacterales*,
- *Pseudomonas* spp.,
- *Acinetobacter* spp.,
- Other Gram-negative aerobic and facultative anaerobic bacteria,
- *Bacteroides* spp.,
- Other anaerobic bacteria, and
- Yeasts.

Other Gram-positive and Gram-negative aerobic and facultative anaerobic bacteria were simply referred to as “other Gram-positive” and “other Gram-negative” bacteria, respectively.

In this study, we use the term ‘incidence’ to refer to the absolute annual number of positive blood cultures reported nationally, without standardisation by population size or hospital activity. Where we report rates per population, this is specified explicitly.

### Covariates

In addition to microbial classifications, we compiled a set of contextual indicators that reflect demographic structure, healthcare activity, and relevant population-level risk factors, in line with the explanatory factors considered in the introduction. These included the mid-year population size, the total number of hospital bed-days, the number of unique inpatients, and the total number of hospital stays recorded each year. We included the number of individuals collecting a prescription for systemic prednisolone as a proxy for immunosuppression. Prednisolone is the most widely used oral corticosteroid in Norway and is prescribed for a broad range of immunosuppressive indications^14^. While this measure does not capture all forms of immunosuppression, it offers a consistent, population-wide indicator based on pharmacy dispensing data. Finally, we included the annual number of incident cancer cases, including a separate count for haematological malignancies and gastrointestinal cancers. The demographic profile was further characterised by the proportion of the population aged 70 years or older.

### Statistical analysis

Contextual indicators and microbial classifications are presented in tables showing values and rates per 100,000 population for 2005 and 2024, together with the percent change over the period. Microbial groups are ordered by Gram reaction and taxonomy, consistent with the NORM surveillance reports.

Missing values were handled pragmatically using either imputation or extrapolation. The number of unique hospitalised patients, which exhibited non-linear temporal dependence, was multiply imputed using chained equations with predictive mean matching. Other time series with partial coverage, specifically the population estimates for the catchment areas of the Central Norway Regional Health Authority (CN) and the Tromsø (T) location of the University Hospital of North Norway, and aerobic bottle counts at Oslo University Hospital, Rikshospitalet in 2005–2007, were extrapolated backwards using linear regression. As UNN earlier had a practice of drawing two aerobic bottles per set, changing to one aerobic bottle per set in 2015, the number of blood cultures used in the denominator were halved in 2005–2014.

The estimated total number of blood cultures taken nationally each year y was then calculated by applying the combined rate of aerobic bottles drawn from CN and T to the total population. Rikshospitalet, Norway’s national referral hospital, was not included in these calculations due to its complex catchment area which reflects its many national functions. Instead, its aerobic bottle counts were used as an external comparison to assess whether the relative trends observed in the estimation also appeared elsewhere. The formula used was

$${Estimated~ total~ blood ~cultures}_{y}={Population~ size}_{y}\times \frac{{Aerobic~ bottles}_{CN,y}+{Aerobic~ bottles}_{T,y}}{{Catchment}_{CN,y}+{Catchment}_{T,y}}$$

allowing the estimated blood culture positivity to be calculated as

$${Estimated~ blood ~culture~ positivity}_{y}=\frac{{Total ~isolates}_{y}}{{Estimated~ total~ blood ~cultures}_{y}}$$

Here, *y* denotes year, *CN* the Central Norway Regional Health Authority, and *T* the Tromsø location of the University Hospital of North Norway.

The uncertainty around the estimated national totals and the positivity indicator was quantified using a non-parametric bootstrap. Counts of isolates and denominator components were resampled under a negative binomial assumption (due to overdispersion), and 95% percentile confidence intervals were derived from the bootstrap distributions.

Temporal trends in the rate distributions of species categories were visualised using line plots. The eight most common species categories in 2024—defined as the top seven by absolute count plus *Streptococcus pneumoniae*—were plotted in one panel, with the remaining species categories shown in a separate panel. Hospital-level trends in blood culture activity were plotted as line graphs with consistent colour mapping, alongside the estimated national total blood culture count. For Oslo University Hospital Rikshospitalet and UNN Tromsø, reconstructed values and hypothetical scenarios were indicated using dashed and dotted lines.

We also plotted the temporal composition of species categories using an area bump chart, illustrating the rank order and relative sizes of the species categories in each year.

To model trends in bacteraemia incidence and positivity, we fitted negative binomial regression models with a log link and an offset for population size, standardised for the proportion of the population aged 70 years or older. Calendar year was included as a restricted cubic spline with four knots to capture non-linear temporal patterns. To investigate the optimal spline fit, three to seven knots were investigated for natural and restricted cubic splines, compared with the Akaike’s Information Criterion (Table S1). Model-predicted incidence and positivity with 95% confidence intervals was plotted alongside the observed incidence.

All analyses were conducted using R version 4.4.0, and the script and full dataset used can be downloaded from GitHub^16^.

## Results

We included a total of 319,149 blood culture isolates from 2005 to 2024, based on data from all microbiological laboratories performing blood cultures in Norway during this period. The Norwegian population increased from 4,606,363 in 2005 to 5,550,203 in 2024, representing a 20.5% growth (Table 1). The mean age increased from 38.7 to 41.3 years (6.7% increase), whereas median age increased from 36.9 to 39.7, and the proportion of the population aged 70 years or older rose from 6.37% to 8.77%, corresponding to a 37.7% relative increase. The sex distribution in the Norwegian population remained relatively stable between 2005 and 2024, with only a slight shift from female predominance to male predominance.

**Table 1:** The absolute numbers and rates per 100,000 people for population characteristics, hospital activity, immunosuppression, cancer incidence, and microbiological testing activity in Norway in 2005 and 2024.

	Absolute numbers			Rate per 100,000 people		
Indicators	2005	2024	% change	2005	2024	% change
Population size	4,606,363	5,550,203	20.5%	-	-	-
Mean age	38.7	41.3	6.7%	-	-	-
Proportion > 70 years old	6.37%	8.77%	37.7%	-	-	-
Hospital bed-days	4,126,986	3,117,434	-24.5%	89,593	56,168	− 37.3%
Hospital stays	958,586	893,266	-6.8%	20,810	16,094	− 22.7%
Unique inpatients	492,203	496,897	1.0%	10,685	8,953	− 16.2%
Prednisolone users (per 1,000)	113,891	209,446	83.9%	2,472	3,774	52.6%
Incident cancer cases	25,494	38,811	52.2%	553	699	26.3%
Incident haematologic cancer cases	2,098	3,457	64.8%	46	62	36.8%
Incident gastrointestinal cancer cases	5,357	7,931	48.0%	116	143	22.9%
Observed blood cultures taken at Rikshospitalet	4,113	9,697	135.8%	-	-	-
Observed blood cultures taken at Ullevål	11,468	14,699	28.2%	-	-	-
Estimated blood cultures taken	187,966	406,150	116.1%	4,081	7,318	79.3%
Estimated blood culture positivity	5.83%	5.58%	-4.3%	-	-	-

As for hospital activity, the rate of hospital bed-days declined from 89,593 to 56,168 per 100,000 population (–37.3%), corresponding to a fall in absolute numbers from 4,126,986 to 3,117,434. This reduction was distributed across hospital stays, which decreased from 20,810 to 16,094 per 100,000 (–22.7%), with absolute numbers falling from 958,586 to 893,266. The number of unique inpatients remained almost unchanged in absolute terms (492,203 vs. 496,897) but declined from 10,685 to 8,953 per 100,000 (–16.2%). According to data from the Norwegian Prescribed Drug Registry, the rate of individuals collecting prednisolone prescriptions increased from 2,472 to 3,774 per 100,000 (52.6%), corresponding to an increase from 113,891 to 209,446 individuals. Incident cancer cases also rose, with overall new cancer diagnoses increasing from 553 to 699 per 100,000 (26.3%; 25,494 vs. 38,811 cases), haematologic cancers from 46 to 62 (36.8%; 2,098 vs. 3,457), and gastrointestinal cancers from 116 to 143 (22.9%; 5,357 vs. 7,931).

Blood culture diagnostics changed over time. The estimated rate of blood cultures taken increased from 4,081 to 7,318 per 100,000 population (79.3%), corresponding to an increase in absolute counts with bootstrapped CIs from 187,966 (95% CI: 127,935–258,803) in 2005 to 406,150 (95% CI: 275,836–556,824) in 2024. At Oslo University Hospital Rikshospitalet, the number of blood cultures taken increased from 4,113 to 9,697 (135.8%), while at Oslo University Hospital Ullevål the increase was from 11,468 to 14,699 (28.2%). Trends in the annual number of blood cultures sampled at selected hospitals and the estimated national total are shown in Supplementary Fig. S1. The estimated blood culture positivity rate with bootstrapped CIs decreased slightly from 5.83% (95% CI: 3.1–10.2%) in 2005 to 5.58% (95% CI: 3.0–9.9%) in 2024, a relative reduction of 4.3%.

Temporal changes in the rates contextual indicators are shown as standardised z-scores in Supplementary Fig. S2, and the correlations between indicators across the study period are shown in Supplementary Fig. S3.

**Fig. 1:**
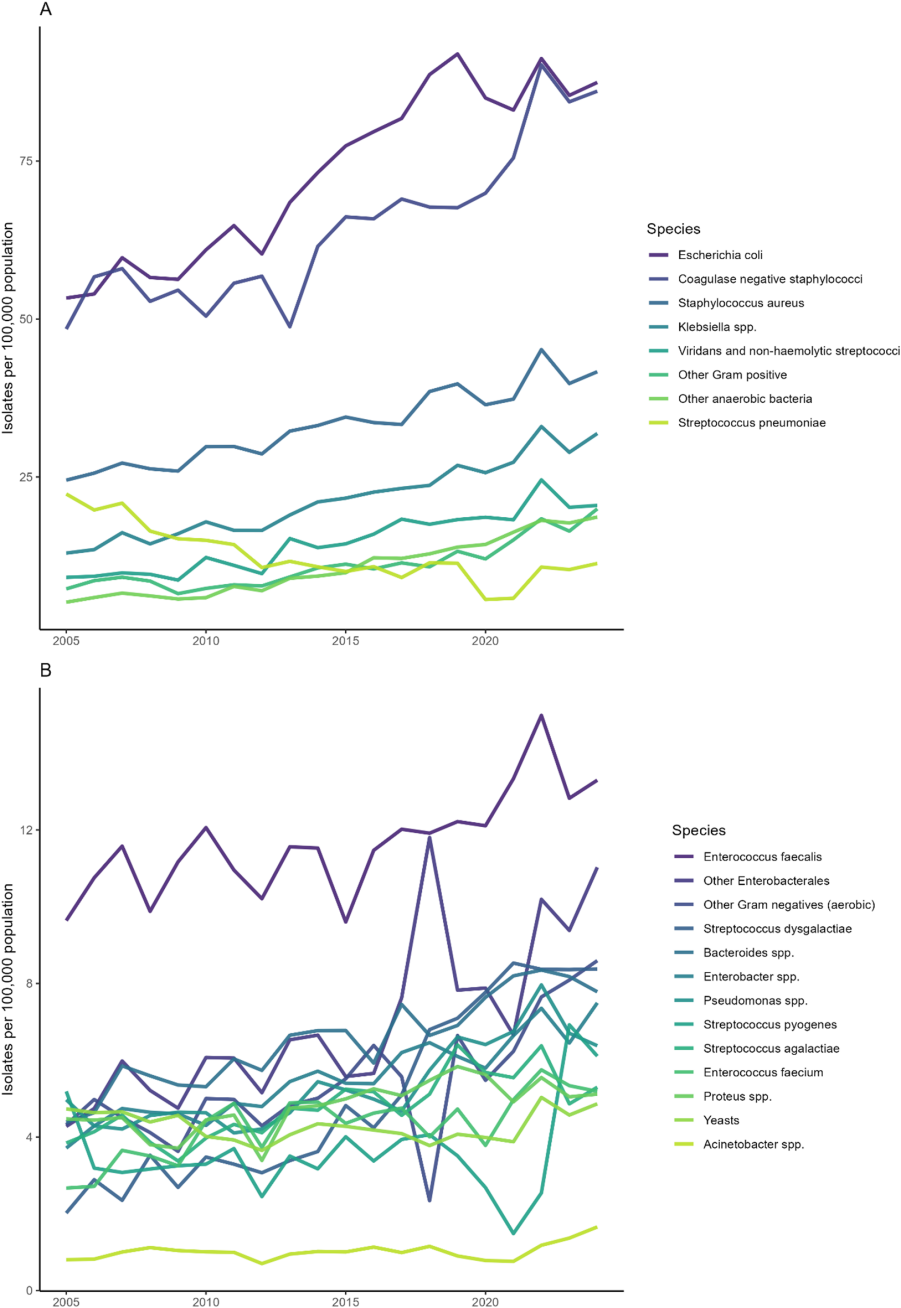
The annual number of blood culture isolates by species category in Norway from 2005 to 2024. The eight most common species in 2024, defined as the seven with the highest absolute count plus *Streptococcus pneumoniae*, are shown in panel A, and all other species categories are shown in panel B.

**Fig. 2:**
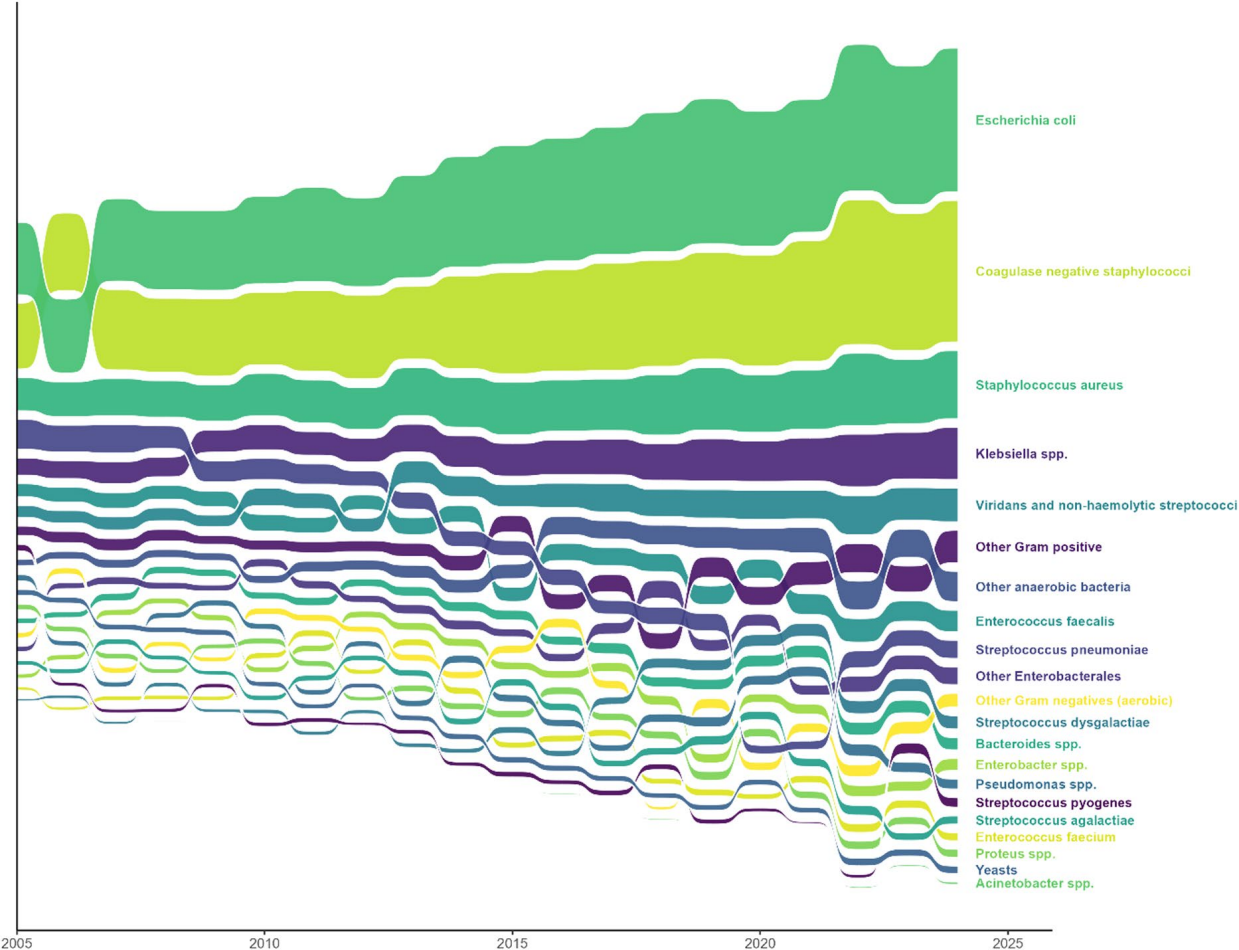
The proportional species distribution among bacteraemia in Norway from 2005 to 2024. The figure shows the relative contribution of each species category to the number of bacteraemia by year, with category proportions represented as stacked bands and their order representing the rank.

*E. coli* was the most frequently identified species throughout the study period, with incidence increasing from 53 to 87 per 100,000 population (64.0%), corresponding to 2,456 isolates in 2005 and 4,854 in 2024 (Table 2, Fig. 1). Another major contributor was *Klebsiella* spp., which rose from 13 to 32 per 100,000 (146.3%; 596 to 1,769 isolates). Several less common Gram-negative species showed similar increases, including *Enterobacter* spp. (101.9%), other *Enterobacterales* (157.8%), and *Acinetobacter* spp. (106.4%). Among Gram-positive species, *S. aureus* increased from 24 to 42 per 100,000 (70.1%; 1,128 to 2,312 isolates), while coagulase-negative staphylococci rose from 48 to 86 per 100,000 (77.8%; 2,230 to 4,777 isolates). Other Gram-positive contributors included viridans and non-haemolytic streptococci (125.2%; 9 to 20 per 100,000) and *Enterococcus faecium* (94.3%; 3 to 5 per 100,000). In contrast, *S. pneumoniae* declined from 22 to 11 per 100,000 population (− 49.3%; 1,027 to 627 isolates). Figure 2 shows the changing species composition across the study period, with a gradual increase in the relative contribution of Gram-negative organisms, largely driven by the decline in *S. pneumoniae*.

**Table 2:** The absolute number and rate per 100,000 people of blood culture isolates by species in 2005 and 2024 and the percentage change between these years. Counts represent the total number of isolates identified from blood cultures in Norway in each year, with relative change shown as percent difference between 2005 and 2024, and the total in bold text.

Species	Absolute counts			Rate per 100,000 people		
	2005	2024	% change	2005	2024	% change
*Staphylococcus aureus*	1,128	2,312	105.0%	24	42	70.1%
Coagulase-negative staphylococci	2,230	4,777	114.2%	48	86	77.8%
*Streptococcus pneumoniae*	1,027	627	-38.9%	22	11	− 49.3%
*Streptococcus pyogenes*	239	339	41.8%	5	6	17.7%
*Streptococcus agalactiae*	177	294	66.1%	4	5	37.9%
*Streptococcus dysgalactiae*	93	465	400.0%	2	8	315.0%
Viridans and non-haemolytic streptococci	419	1,137	171.4%	9	20	125.2%
*Enterococcus faecalis*	444	738	66.2%	10	13	38.0%
*Enterococcus faecium*	123	288	134.1%	3	5	94.3%
Other Gram-positive	335	1,107	230.4%	7	20	174.3%
*Escherichia coli*	2,456	4,854	97.6%	53	87	64.0%
*Klebsiella* spp.	596	1,769	196.8%	13	32	146.3%
*Enterobacter* spp.	171	416	143.3%	4	7	101.9%
*Proteus* spp.	206	284	37.9%	4	5	14.4%
Other* Enterobacterales*	197	612	210.7%	4	11	157.8%
*Pseudomonas* spp.	229	354	54.6%	5	6	28.3%
*Acinetobacter* spp.	37	92	148.6%	1	2	106.4%
Other Gram-negative	199	477	139.7%	4	9	98.9%
*Bacteroides* spp.	202	432	113.9%	4	8	77.5%
Other anaerobic bacteria	238	1,035	334.9%	5	19	260.9%
Yeasts	218	270	23.9%	5	5	2.8%
**Total**	**10,964**	**22,679**	**106.8%**	**238**	**409**	**71.7%**

**Fig. 3:**
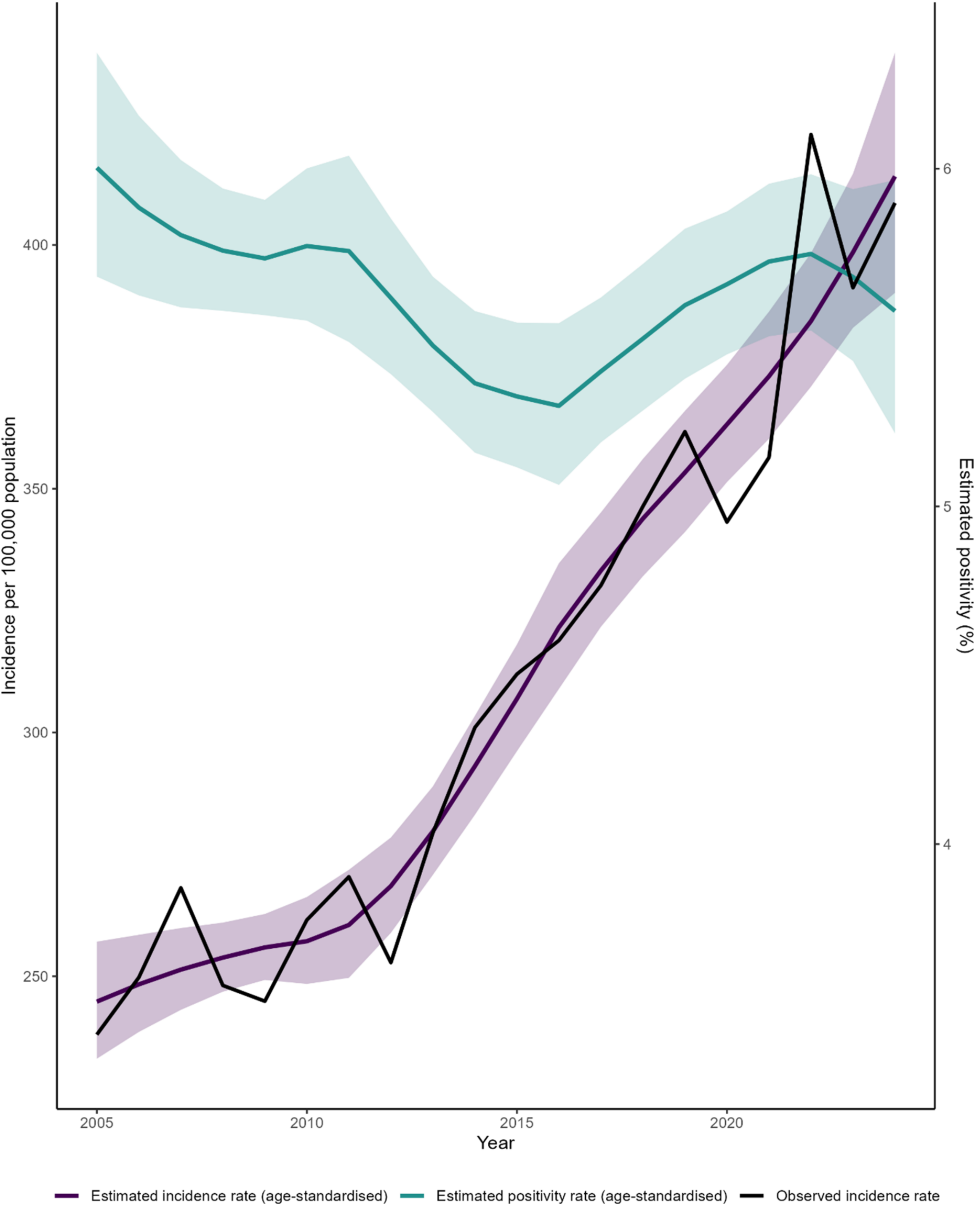
Observed and modelled annual incidence of bacteraemia and estimated blood culture positivity in Norway, 2005–2024. The black line shows the observed incidence per 100,000 population. The purple line shows fitted values from a negative binomial regression model of incidence, adjusted for the proportion of the population aged 70 years or older, with shaded areas representing 95% confidence intervals. The blue-green line shows fitted values from a corresponding age-adjusted model of blood culture positivity, expressed as a percentage, with shaded areas representing 95% confidence intervals.

Our main regression models were age-standardised. Figure 3 shows the observed incidence of bacteraemia alongside modelled age-standardised estimates with 95% confidence intervals, together with a corresponding model of blood culture positivity.

## Discussion

In this ecological study of bacteraemia in Norway from 2005 to 2024, the incidence of bacteraemia increased both in absolute numbers and per 100,000 population. The increase coincided with demographic ageing, rising immunosuppression and cancer incidence, and more than a doubling in the number of blood cultures taken, while the number of hospital admissions per capita declined. *E. coli* remained the most common pathogen, followed by *S. aureus* and *Klebsiella* spp., all of which increased substantially over time. In contrast, *S. pneumoniae* declined. Regression models showed a steady rise in incidence over the study period, with minimal difference between unadjusted and age-adjusted estimates.

Our findings align with those from other Nordic countries reporting on national or larger regional trends in the aetiology of bacteraemia. Dessau et al. found a marked increase in the incidence of positive blood cultures in Denmark between 2010 and 2022, alongside a 64% increase in blood culture sampling and a relatively stable positivity rate around 10%, arguing that this may be linked to demographic shifts with a higher proportion of elderly^4^. Two studies from Sweden and Finland similarly reported an increasing incidence of Gram-negative bacteraemias between 2000 and 2014, with *E. coli* and *Klebsiella* spp. as the main contributors and a decline in pneumococcal bacteraemia, closely matching our observations^5,6^. An earlier Norwegian study covering 1999–2008 also documented that bacteraemia were increasing in incidence already then^3^. Outside the Nordic countries, comparable population-based studies are rare. As examples of the scarcity of nationwide data outside of the Nordic region, a Spanish study from 2010 to 2019 reported a lower overall incidence, but the same increase in Gram-negative bacteraemias and decrease in *S. pneumoniae*, but was limited to two hospitals in Madrid^17^, while a single-centre study from Vietnam demonstrated a strikingly high relative incidence of idiosyncratic microbes like the porcine-associated *Streptococcus suis* and the non-fermenter *Stenotrophomonas maltophilia*^18^. Together, these comparisons suggest that a rising overall incidence and a shift towards certain Gram-negative pathogens may represent a broader phenomenon, at least in the Nordic countries though differences in study design, scope, and data completeness complicate direct comparisons.

In our study, the species category distribution among the blood culture isolates also changed over the study period. The most notable decline was observed for pneumococcal bacteraemia. This trend likely reflects the introduction and scale-up of childhood pneumococcal conjugate vaccination programmes in Norway. Similar declines has been reported from other high-income countries following vaccine implementation^19,20^. In contrast, most other organisms showed marked increases. In other words, the apparent rise in Gram-negative bacteria relative to Gram-positives (not including contaminants) is largely explained by the decline in *S. pneumoniae*. Within *Enterobacteriaceae*, the incidence of *E. coli* bacteraemia roughly doubled, while *Klebsiella* spp*.* tripled, underlining the growing importance of these organisms in the epidemiology of bacteraemia. The relative increase in the *Klebsiella* spp*.* to *E. coli* ratio has also been noted in European-level surveillance data, suggesting a wider trend beyond Norway ^21^. Although a specific association with nosocomial infections is not confirmed by a previous large Norwegian study^22^, *Klebsiella* spp. infections are often considered more weighted towards hospital settings than *E. coli.* A similar distinction applies between Enterobacterales and non-fermenters such as *Pseudomonas* spp. and *Acinetobacter* spp., whose proportional changes can serve as ecological indicators of hospital-associated infection patterns^23^. While the absolute incidence was low, the relative increase in *Acinetobacter* spp. was also notable towards the end of the study period, in contrast to *Pseudomonas* spp., whose relative importance decreased. While a major increase in extensively drug-resistant *Acinetobacter* spp. has been reported from other European countries^24^, the increase we observe in Norway does not reflect resistant strains. All carbapenemase-producing *Acinetobacter* spp. are notifiable to the national surveillance system, and no marked rise has been recorded among blood culture isolates. Notably, *S. dysgalactiae* and anaerobes showed some of the steepest relative increases. The *S. dysgalactiae* category, comprising β-haemolytic group C and G streptococci, has been identified using consistent criteria despite methodological changes, with MALDI-TOF introduced and routinely implemented by around the middle of the study period. *S. dysgalactiae* has been recognised as an emerging cause of invasive infection in recent years, and improved diagnostics for anaerobes may also have contributed to their observed increase^25^. Increases were also seen in viridans and non-haemolytic streptococci, as well as in the “other” categories for both Gram-positive and Gram-negative organisms, potentially indicating an increased ecological diversity in the aetiology of bacteraemia. These patterns may suggest a growing contribution of healthcare-associated or hospital-onset infections as these are sometimes low-virulence or rare microbes that may cause disease in the frail or immunosuppressed, consistent with changes in patient case-mix and invasive procedures^26,27^. However, our ecological design does not allow for causal, patient-level attribution. National guidelines and laboratory routines for blood culture sampling remained stable throughout the study period, making it unlikely that such changes influenced the observed trends. Furthermore, although Norway has a programme for the medical evacuation of war casualties in Ukraine^28^, this cannot account for the observed increase, as the programme only began after 2022 and the number of patients transferred has been limited to the double digits.

These microbial shifts occurred during a period when antibiotic consumption in hospitals remained relatively stable in overall volume, with a gradual move towards narrower-spectrum agents and more targeted prescribing^10^. While total number of defined daily doses (DDDs) per population changed little before the COVID-19 pandemic, antibiotic use adjusted for hospital activity (e.g. bed-days) showed considerable variation between hospitals and years. Broad-spectrum antibiotics such as cephalosporins, fluoroquinolones and carbapenems made up a decreasing share of use, concurring with an increased use of aminoglycosides and glycopeptides, suggesting strengthened stewardship. In primary care—where more than four-fifths of all antibiotics are prescribed—overall use declined between 2012 and 2019, mainly due to reduced prescribing for respiratory tract infections. Although there was a rebound after the COVID-19 pandemic, levels in 2023 were still comparable to those seen in 2019 and well below earlier years. The prescribing pattern has remained dominated by narrow-spectrum agents, particularly phenoxymethylpenicillin, pivmecillinam, dicloxacillin, amoxicillin, doxycycline, and nitrofurantoin, with broad-spectrum antibiotics making up a relatively small and decreasing share of total prescriptions^10^. Taken together, these developments suggest that changes in antimicrobial use are unlikely to have been the main driver of the observed increase in bacteraemias or the shift towards a higher contribution of Gram-negative organisms, although a contributory role cannot be excluded. The observed increases in incidence among some species may have important implications for infection prevention and control in several different ways^29^. Increasing incidence of species inherently resistant to certain antibiotics may challenge established empirical treatment regimens. Also, these species may harbour antimicrobial resistance genes on mobile genetic elements, facilitating horizontal transmission between bacterial species, which may further exacerbate the threat of acquired antimicrobial resistance in the longer term. Finally, some of these species have been found in hospital water systems, which—regardless of whether they serve primarily as reservoirs or recipients—highlight the need for attention to environmental hygiene and standard precautions in healthcare settings.

A general demographic trend in high-income countries is declining fertility rates and an ageing population, which together are reshaping both population structure and healthcare expenditure^30^. The proportion of Norwegians aged 70 years or older increased by 38% during the study period, reflecting this broader pattern. In parallel, due to resource constraints, the healthcare system is under pressure to become more efficient, driven by a shrinking working-age population relative to the number of individuals requiring care. This shift is evident in our data, where the number of hospital bed-days declined by nearly a quarter despite a largely stable number of hospital stays. This suggests a trend towards shorter lengths of stay and higher patient turnover. In parallel, advanced outpatient services, including so-called “hospitals at home”, are expanding. Much of the apparent reduction in hospital activity per patient likely reflects a redistribution of care, with patients more often discharged early to municipal services that now provide an increasing volume of hospital-like healthcare. Together with an ageing population, this may result in a relatively sicker inpatient population with more complex diagnostic panoramas^31^. In our data, this was reflected in a steeper rise in bacteraemia incidence when expressed per bed-day than when expressed per population. Additionally, markers of immunosuppression, such as the number of individuals collecting prednisolone prescriptions and the number of incident cancer cases, increased substantially over time, both of which are recognised risk factors for bloodstream infections. Prednisolone use represents only one part of a broad spectrum of causes of immunosuppression and likely underestimates the overall burden. However, gastrointestinal cancers, particularly associated with infections due to Gram-negative bacteria, did not increase more than all cancers combined. Furthermore, age has been demonstrated to be associated with shifts in the epidemiology of bloodstream infections, with the relative importance of different pathogens varying across age groups^32^. Demographic ageing and increasing morbidity, including higher levels of immunosuppression, are likely contributing to the rise in bacteraemias, but our simple adjustment for the proportion aged 70 years or older does not capture these underlying changes. More detailed individual-level data would be needed to clarify their relative impact.

Diagnostic intensity increased substantially during the study period, as reflected by our estimated doubling of national blood culture sampling. Although these estimates rely on extrapolations from data in the Central Norway Regional Health Authority and the University Hospital of North Norway, Tromsø, a similar temporal pattern observed at Oslo University Hospital—which was not included in the national estimate due to its complex catchment area and national functions—supports the plausibility of a nationwide increase in sampling. While absolute numbers are uncertain, the direction of change appears robust across data sources. Importantly, although our approach does not provide a valid estimate of the absolute blood culture positivity rate, the relative stability of the estimated indicator over time, in line with findings from other Nordic countries^33^, argues against a major shift towards lower-yield or more indiscriminate testing practices. This impression is reinforced by the stable proportion of coagulase-negative staphylococci among all isolates, which accounted for 20% of all isolates in 2005 and 21% in 2024. High quality data on contamination rates cannot be obtained without a prospective design with harmonised criteria across hospitals and laboratories. In their absence, the two indicators we use suggest that the rise in positive blood cultures primarily reflects a real increase in the underlying burden of bloodstream infections, rather than increased contamination or a systematic lowering of the threshold for sampling.

A major strength of this study is the use of a complete national dataset covering all microbiology laboratories performing blood cultures in Norway over a 20-year period. This allowed for an unselected, population-wide analysis with long-term trend data. However, several limitations must be acknowledged. The ecological design precludes causal inference, and we cannot definitively establish whether the observed increase in positive blood cultures represents a true increase in the incidence of bloodstream infections or is driven by increased diagnostic activity, changing indications for blood culture sampling, or improved detection and identification methods. We lack individual-level data on patient characteristics, blood culture indications, sampling rates, and timing relative to hospital admission, precluding analyses that could distinguish between community- and hospital-onset infections or calculate incidence per patient-days at risk. The denominator for total blood cultures taken was estimated based on data from selected regions, and the Oslo hospitals were excluded from this calculation due to their complex catchment areas and extensive national referral functions, which result in an atypical patient mix (*Rikshospitalet*, literally ‘the national hospital’). Although both Rikshospitalet and Ullevål also provide local and regional services, their activity profiles differ substantially due to variations in patient composition and specialist functions. These differences, rather than regional variation in diagnostic practice, might explain the divergence observed between the Oslo hospitals and other sites. However, the denominator was used mainly to capture the trend in diagnostic activity over time rather than to estimate the absolute number of cultures taken. Although trends at these hospitals support our national estimate, relying on data from only Central and Northern Norway introduces uncertainty. If blood culture activity in other regions followed different patterns, our estimates of national sampling volumes—and thus of positivity—could be biased in either direction. Furthermore, we were unable to fully consider possible changes in contamination rates, although the stability of the proportion of coagulase-negative staphylococci suggest no major changes in blood culture quality. Finally, we relied on aggregated species categories, which limits species-specific interpretations. We did not attempt to model or test statistical associations between incidence and the contextual factors we present. The incidence of bacteraemia increased monotonically and nearly linearly over the study period, and such a secular trend will inevitably correlate with any other factor displaying a steady rise, such as population size, population age, GDP, or even unrelated metrics like food consumption. In this setting, ecological correlations are therefore not informative and risk being misleading, and we considered such analyses to add little value to the interpretation. Future work should prioritise person-level studies with linked clinical, microbiological, and administrative data to better understand and estimate the relative impacts of the drivers behind these trends, distinguish between hospital- and community-onset infections, and explore patient-level risk factors. Improved national surveillance with denominator data on blood cultures taken, hospital admissions, and patient-days could also strengthen future analyses.

In conclusion, the incidence of bacteraemia in Norway increased steadily between 2005 and 2024, accompanied by marked shifts in microbial composition towards Gram-negative organisms. This increase occurred alongside demographic ageing, rising immunosuppression, and increased diagnostic activity, but remained evident after adjusting for population age structure. Although changes in testing practices may have contributed, the stability of the positivity rate and the proportion of likely contaminants suggest that the observed increase reflects a genuine rise in bacteraemia. These findings provide a national reference for bacteraemia trends over two decades and highlight the need for continued surveillance and more detailed, individual-level research to better understand the drivers of these changes.

## Supplementary Information

Below is the link to the electronic supplementary material.

Supplementary Material 1

## Data Availability

All data used in this article may be found in the referenced repository (https://github.com/andersskyrud/bacteraemia_species/).
