# Supplementary material for: Two decades of bacteraemia in Norway: an ecological study of incidence and shifts in microbial epidemiology, 2005–2024
Source: Sci Rep. 2025 Dec 29;15:44870. doi: 10.1038/s41598-025-28472-x (PMC12749306; doi:10.1038/s41598-025-28472-x)
Supplement: Supplementary file 1 — Supplementary Material 1 [file 41598_2025_28472_MOESM1_ESM.docx]

# Supplementary materials


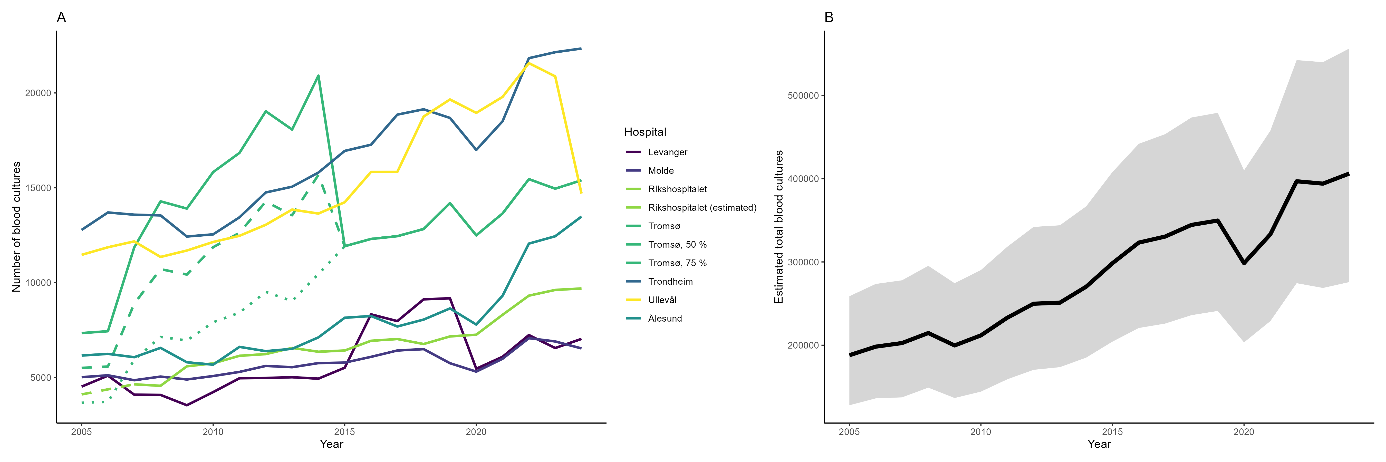


Figure S1. Annual number of blood cultures taken at selected hospitals (panel A) and the estimated total number of blood cultures taken (panel B) including bootstrapped confidence intervals in Norway, 2005–2024. For UNN Tromsø, dashed and dotted lines represent 75% and 50% of the count, respectively, to account for periods with additional aerobic bottles. For Oslo University Hospital Rikshospitalet, the dashed line represents reconstructed values for years with incomplete data.


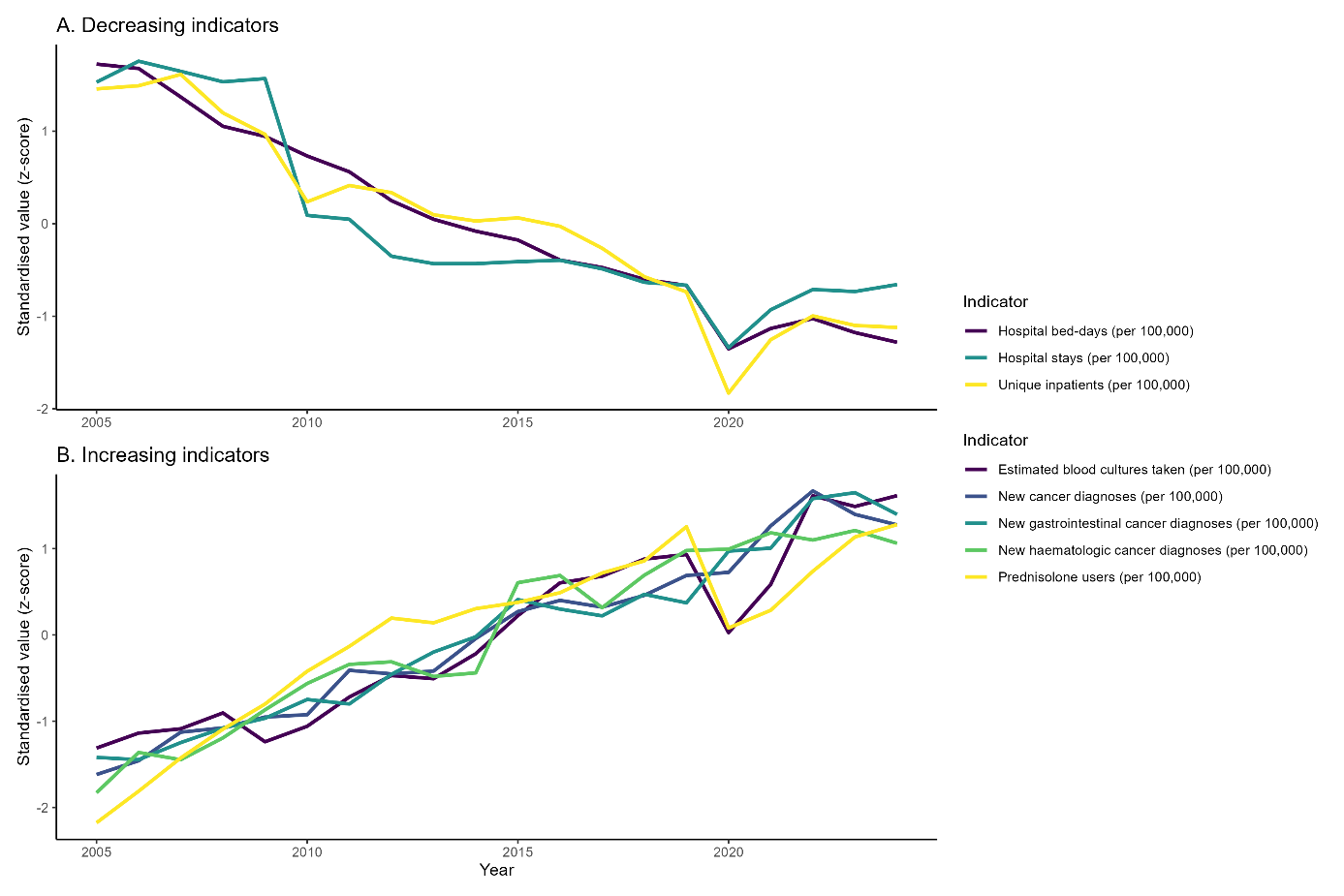


Figure S2. Standardised temporal trends in contextual indicators in Norway, 2005–2024. Panel A shows indicators with decreasing rates, and panel B shows indicators with increasing rates. Lines represent standardised (z-score transformed) annual values relative to the mean and standard deviation across the study period. All indicators are expressed as rates per 100,000 people. Indicator names are shown in the legend.


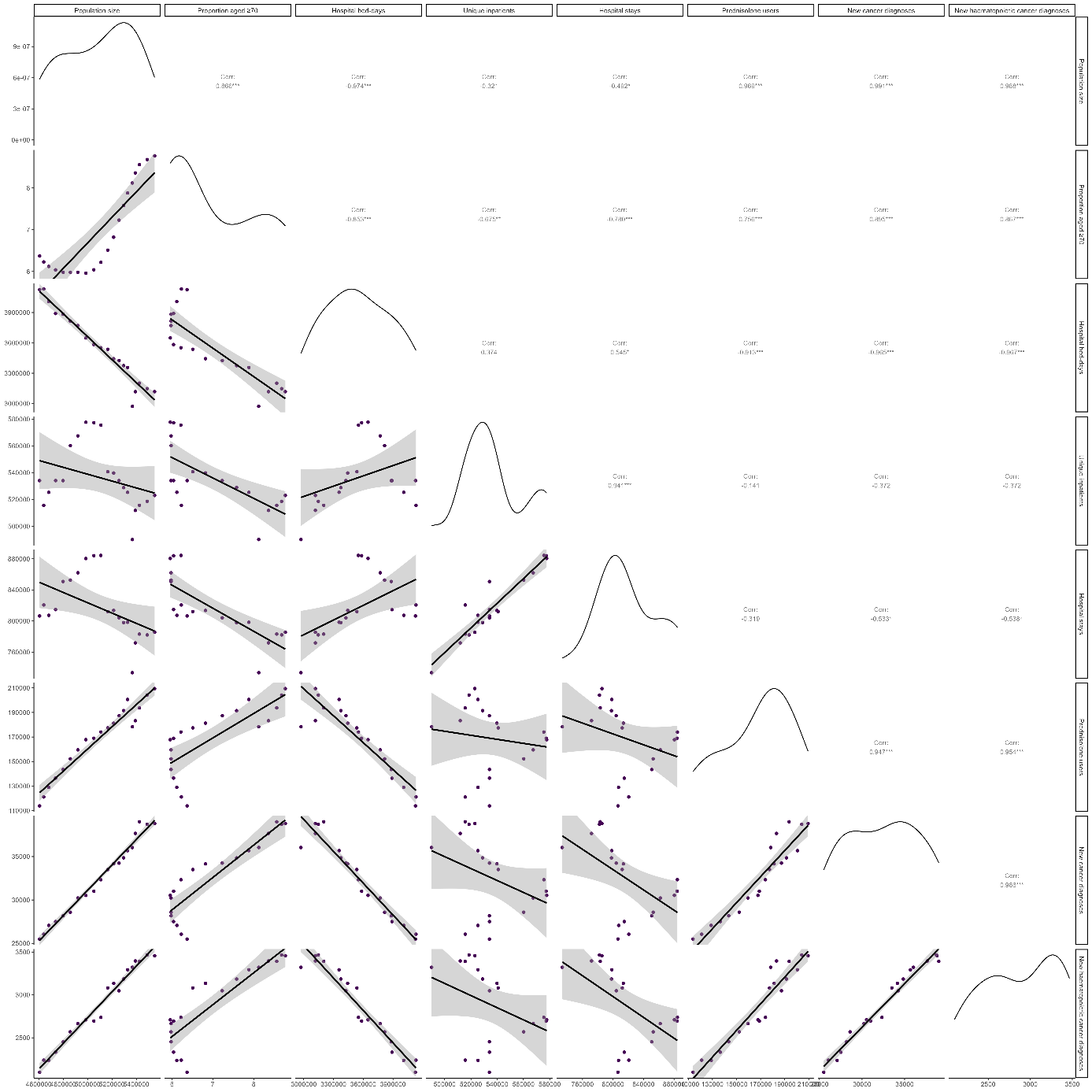


Figure S3. Pairwise correlations between contextual indicators in Norway, 2005–2024. The lower panels show scatterplots with fitted smooth lines and 95% confidence bands for each indicator pair. The upper panels display Pearson correlation coefficients with significance levels, and the diagonal panels show the distribution of each indicator as density plots. Indicator names are shown along the axes.


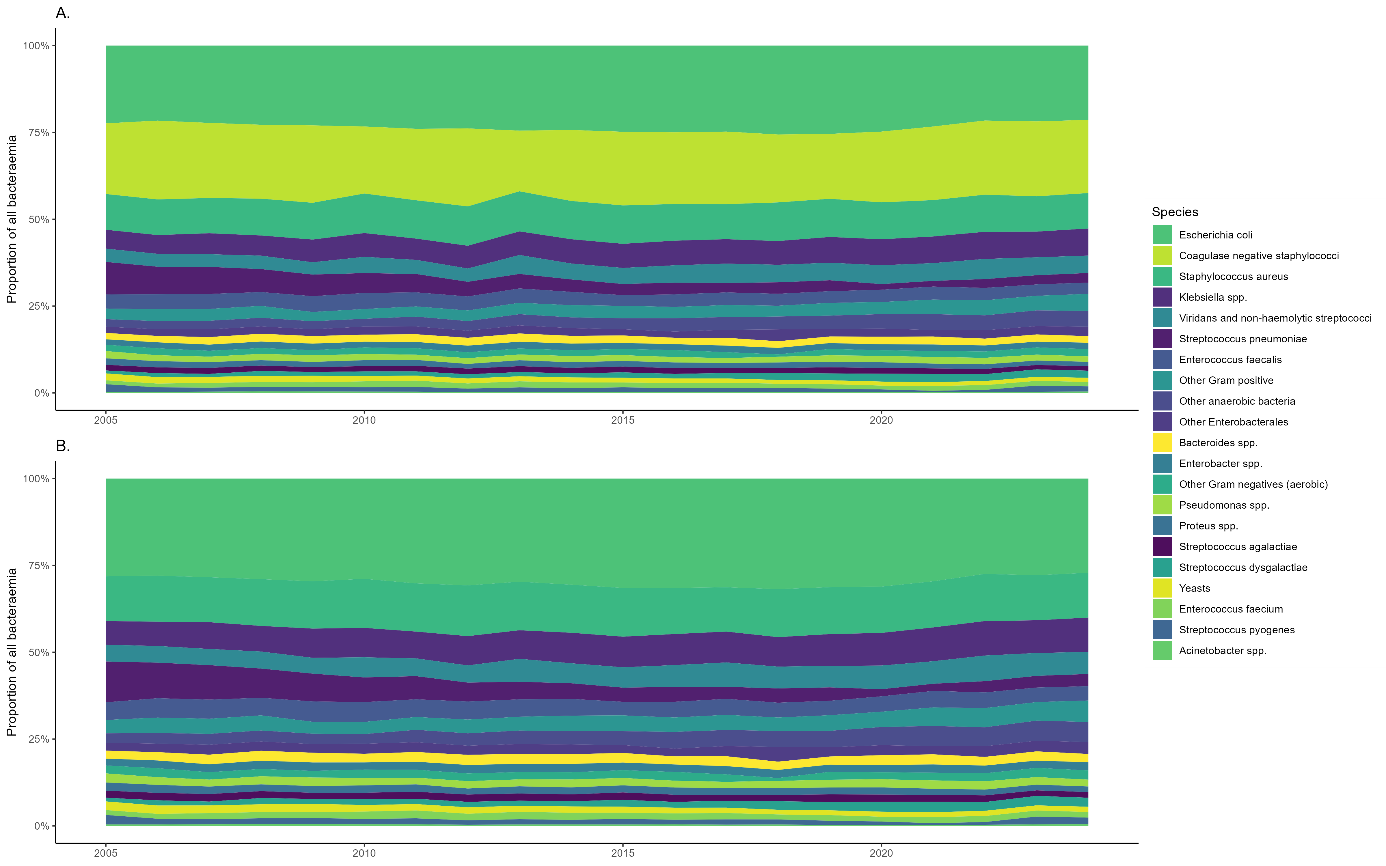


Figure S4. Distribution of microbial species among bacteraemia isolates in Norway, 2005–2024. Panel A shows the proportional distribution of all species categories within 100% of isolates. Panel B shows the proportional distribution excluding coagulase-negative staphylococci, to illustrate relative changes among the remaining species.

Table S1. Akaike’s Information Criterion (AIC) values for natural and restricted cubic spline models with varying numbers of knots.

| **Natural splines** | | **Restricted cubic splines** | |
| --- | --- | --- | --- |
| **Knots** | **AIC** | **Knots** | **AIC** |
| 2 | 325.9681 | - | - |
| 3 | 323.7153 | 3 | 326.1541 |
| 4 | 325.8532 | 4 | 322.9792 |
| 5 | 326.0295 | 5 | 324.5502 |
| 6 | 327.0994 | 6 | 325.7999 |
| - | - | 7 | 327.3492 |
